# Performance of the *UroVysion* ^*®*^ FISH assay for the diagnosis of malignant effusions using two cutoff strategies

**DOI:** 10.1002/cam4.1442

**Published:** 2018-03-25

**Authors:** Débora C. B. Rosolen, Daniel K. Faria, Caroline S. Faria, Leila Antonangelo

**Affiliations:** ^1^ Division of Clinical Pathology Department of Pathology Faculdade de Medicina Hospital das Clinicas HCFMUSP Universidade de Sao Paulo Sao Paulo SP Brazil; ^2^ Faculdade de Medicina Medical Investigation Laboratory (LIM03) Hospital das Clinicas HCFMUSP Universidade de Sao Paulo Sao Paulo SP Brazil

**Keywords:** Aneuploidy, ascites, cytology, fluorescent in situ hybridization, malignancy, pleural effusion

## Abstract

The cytological examination of cavity fluids has limited sensitivity in the diagnosis of malignancy. Aneuploidy, which is commonly observed in neoplastic cells, could potentially be used as an ancillary diagnostic tool. To evaluate the detection of aneuploid cells in cavitary effusion samples using the fluorescence in situ hybridization (FISH) assay *UroVysion* ^®^ with some adaptations and two different cutoff strategies. Seventy samples of pleural or peritoneal fluid with positive (*n* = 40), negative (*n* = 15), or suspicious (*n* = 15) oncotic cytology were subjected to FISH assay with the multitarget *UroVysion* ^®^ kit, which is composed of probes that hybridize to the centromeric region of chromosomes 3, 7, and 17 and to the locus 9p21. FISH performance was evaluated using two different cutoffs: (1) the manufacturer's cutoff (*M‐FISH*) and 2) a proposed cutoff (*P‐FISH*). Using *M‐FISH*, the diagnostic sensitivity was 57.1%, specificity 87.5%, and accuracy 60.0%; with *P‐FISH*, the sensitivity was 87.3%, specificity 71.4%, and accuracy 85.7%. When combined with cytology, the sensitivity, specificity, and accuracy were 88.0%, 83.3%, and 87.8%, respectively. Malignant cells presented a predominance of chromosomal gains. The *UroVysion* ^®^ test using the *P‐FISH* cutoff was effective in demonstrating aneuploid cells in all malignant effusions, confirming the diagnosis of malignancy even in cases with suspicious cytology.

## Introduction

The accumulation of fluid in serous cavities can represent a systemic complication or local disease 1. When systemic diseases progress with effusion, the diagnostic and therapeutic approach is usually restricted to the identification and treatment of the underlying cause, such as a pleural effusion associated with congestive heart failure 1 or ascites associated with cirrhosis and portal hypertension 2. In contrast, local involvement of the pleura and/or peritoneum requires a precise and usually multilaboratory typically intersectional diagnosis.

Malignancy comprises one of the leading causes of exudative cavity effusion 3. It is estimated that approximately 100,000 to 150,000 individuals per year present malignant pleural effusion in the United States and Europe, of which 50–65% are secondary to lung and breast cancers 4. Although guided biopsy is the gold standard for demonstrating neoplastic serous involvement, this procedure is not always feasible due to the clinical condition of most patients with advanced disease and the expense involved 5.

Cytological analysis of pleural and/or peritoneal fluid obtained by aspiration is the first step in the diagnosis of malignant effusions. The sensitivity of this method varies from 60% to 96% depending on the type and location of the tumor, the techniques used for preparation and staining, and the cytologist's expertise in identifying malignant cells 2, 6, 7.

Thus, cytological examination does not provide a definitive diagnosis in up to 40% of cases. Ancillary techniques using samples obtained by aspiration puncture—a procedure considered minimally invasive and of low‐risk—are therefore recommended to improve diagnosis. Diagnostic tools explored in recent decades include the quantification of liquid‐soluble tumor markers 7, immunocytochemistry in embedded materials 8, DNA ploidy analysis by flow cytometry 9, and molecular assays such as polymerase chain reaction (PCR) 10.

Aneuploidy is a common finding in neoplastic cells 11, and the demonstration of abnormal cell DNA content is considered indicative of malignancy. Fluorescence in situ hybridization (FISH) has been used in cavity fluids to detect aneuploidy in interphase cells, circumventing the need for cell culture, which could delay the turnaround time (TAT) to result 12, 13.

This study proposes to evaluate the detection of aneuploid cells in pleural and peritoneal fluid samples using the *UroVysion®* test, originally developed for the diagnosis of bladder cancer 14. To analyze the diagnostic performance of *UroVysion®,* we used two different cutoff strategies: (1) the manufacturer cutoff (*M‐FISH*) and (2) a proposed cutoff (*P‐FISH)* developed in this study.

## Materials and Methods

Seventy patients with cavitary effusion (pleural and peritoneal) who had been admitted to the *Hospital das Clinicas da Faculdade de Medicina da Universidade de Sao Paulo* (HC‐FMUSP) were included in the study after providing informed consent. Each sample was representative of one patient. The fluid samples were submitted to conventional biochemistry, microbiology, and cytology examinations for diagnostic evaluation. Routine tests were performed by the HC‐FMUSP clinical laboratory, which is accredited by the *College of American Pathologists* (CAP). The study was approved by the institutional ethics committee.

The variables analyzed were age, gender, and clinical diagnosis according to the *International Statistical Classification of Diseases and Related Health Problems (CID), 10th Revision of Codes*. Clinical and laboratory data were extracted from medical records and the laboratory system database, respectively. Histopathological diagnosis was considered the gold standard for malignancy. In benign cavitary effusions, clinical history, laboratory and imaging examinations, and patient follow‐up were used to exclude malignancy.

### Cytological examination

**Figure 1:**
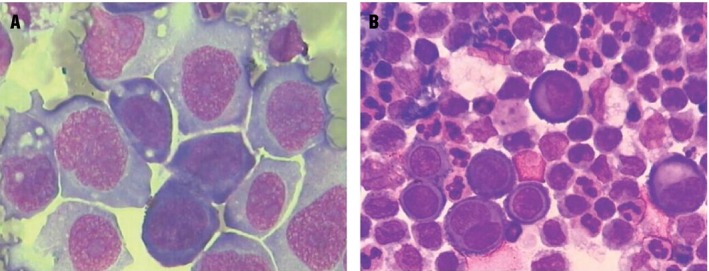
Cytological characteristics of a malignant and a reactive pleural fluid sample. (A) Tumor cells clustering in case of malignant pleural effusion (Leishman); (B) reactive mesothelial clustering of cells in benign pleural effusion (Leishman).

After macroscopic sample analysis, nucleated cells were counted in a counting chamber, and the fluid samples were centrifuged (2000 rpm, 10 min) to prepare the slides. Cytological examination (cell differentiation and oncotic cytology) was performed on slides stained with hematological dye (Leishman stain) (Fig. 1).

Based on oncotic cytology, the cases were classified into three categories: “positive,” “suspicious,” or “negative”. In this study, the following conditions were considered “concordant”: (1) “suspicious” or “positive” oncotic cytology and “positive” gold standard; or (2) “negative” oncotic cytology and “negative” gold standard. *Light's criteria* 15 for pleural fluid and Rovelstad et al. 16 for peritoneal fluid were used to classify the samples in exudates or transudates.

### Molecular cytogenetic study

For cytogenetic analysis, the samples were treated using the commercial multitarget *UroVysion* FISH kit (Abbott, IL, cat. nº 32‐161070) with centromeric alpha probes for chromosomes 3 (*CEP* ^*®*^ *3 Spectrum red*), 7 (*CEP7 Spectrum Green*), 17 (*CEP17 Spectrum Aqua*), and the locus‐specific probe 9p21 (*LSI* ^*®*^ *p16 Spectrum Gold*). The sample was centrifuged (1400 rpm, 5 min), and the pellet obtained was fixed with fresh *Carnoy* (methanol/acetic acid solution, 3:1 ratio) for slide preparation. The slides were subsequently hybridized with centromeric probes for chromosomes 3 (red‐labeled), 7 (green‐labeled), 17 (blue‐labeled), and the 9p21 region (yellow‐labeled). The manufacturer's instructions were followed with slight modifications to timing and temperature. The digital images obtained were captured on an *Olympus BX41 microscope* equipped with a 100 W lamp and fluorescein filters for propidium iodide (FITC‐PI, BP 450‐490, FI 510, and BP 520, Cat # 487709). The Applied Imaging *CytoVision System* (San Jose, CA) was used to analyze the images.

A total of 200 interphase cells per sample were analyzed, and only cells with clearly distinguishable signals for monosomy and/or polysomy were counted. A cell was considered aneuploid when marked by the loss or gain of at least two probes (3, 7, or 17) with or not the loss of the 9p21. In the absence of two signals for chromosomes 3, 7, and 17, the cells were considered noninterpretable. Because benign reactive mesothelium can present tetraploidy, cells with these characteristics were excluded from the analysis. This criterion was previously used by Rosolen et al. 13 and Flores‐Staino et al. 17 in similar work. The slides were evaluated by two independent observers, and the results represent the average of their measurements.

**Figure 2:**
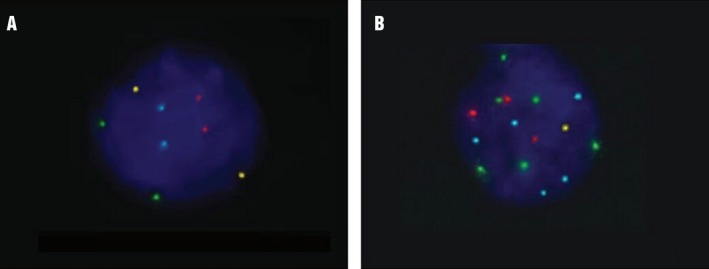
FISH images showing a euploid cell in a benign peritoneal effusion case (A) and an aneuploid cell in a malignant pleural effusion case (B). A. Pleural effusion cells showing euploid cells (2n) for chromosomes 3 (red), 7 (green), 17 (blue), and 9p21 (yellow); FISH, UroVysion, 1000×; B. pleural effusion cells showing aneuploidy for chromosomes 3 (red), 7 (green), 17 (blue), and 9p21 (yellow); FISH, UroVysion, 1000×.

To classify a case as aneuploid, it is suggested that each laboratory establish its own cutoff for the genetic changes observed. Thus, an effusion was considered aneuploid when the number of abnormal cells was higher than the previously established cutoff (Fig. 2). To establish this value, we analyzed the diploid (normal) and nondiploid (abnormal) signals emitted by cells present in fluid obtained from patients with a known benign effusion. So, for the analytical validation of probe parameters and results interpretation, we used the statistical test of the inverse *β* function (probability in decimal), where *α* = 1 + *X* (*X* represents the highest number of positive signals obtained by the observers) and *β *= number of cells analyzed 18. *P‐FISH* for the four probes were as follows:

1. Chromosome 3: >3.0% for one signal or >3.0% for three or more signals;
2. Chromosome 7: >4.0% for one signal or >2.0% for three or more signals;
3. Chromosome 17: >4.0% for one signal or >3.0% for three or more signals;
4. Chromosome 9p21: >4.6% for one signal.

According to the manufacture's criteria (counting 25 cells), cells were classified as aneuploid when presenting ≥ 4 gains in the same cell for two or more chromosomes (3, 7, or 17) or ≥ 12 cells with zero signal for 9p21. The performance of the *UroVysion®* FISH test was calculated according to the two cutoffs (*M‐FISH* and *P‐FISH*).

### Statistical analyses

**Figure 3:**
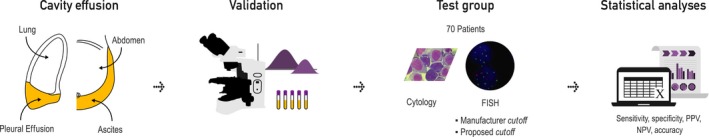
Summary of the study design.

Continuous variables are described by the median, mean, and standard deviation (SD). Categorical variables are presented as a percentage. A comparison of the performance of *UroVysion* ^*®*^ FISH with both cutoffs was made by chi‐square test or Student's *t* test. The significance level *P* < 0.05 was adopted. Contingency analyses were performed to determine sensitivity, specificity, positive predictive value (PPV), negative predictive value (NPV), and accuracy. Data were analyzed using the *Microsoft Office 365 Excel program*s (Redmond, WA) and the *17th version of Minitab Statistical Software* (Minitab Inc. International Sales and Support, State College, PA). Figure 3 summarizes the study design.

## Results

Seventy patients with pleural or peritoneal effusion were included: 63 (90%) with malignant etiology and seven (10%) of benign origin. From the malignant effusions, positive cytology was observed in 40 (63.5%), negative in nine (14.3%), and suspicious in 14 (22.2%) cases. The general characteristics of the study group and of the fluids are shown in Table 1.

**Table 1:** Characteristics of the study group and of the fluid samples

Patients (*N*)	70
Age	
Mean ± SD	62.8 ± 15.0
Median	63.0
Male/Female (*N*)	26/44
Pleural/Peritoneal (*N*)	60/10
Aspect	
Before centrifugation	
Yellow/Ser‐H/Hemorrhagic/Brownish/Colorless/Purulent	37/17/13/1/1/1
After centrifugation	
Yellow/Erythrochromic/Brownish/Colorless/Purulent	65/3/1/1/0
Color	
Before centrifugation	
Clear/slightly cloudy/cloudy	2/11/25
After centrifugation	
Clear/slightly cloudy/cloudy	65/2/2
Cell Count (mm^3^) Median	755
Neutrophils/Lymphocytes predominance (*N*)	21/49
Cytology	
Positive/suspicious/negative	40/15/15
Transudate/Exudate (*N*)^a^	15/45
ADA ± SD	11.4 ± 9.3
Positive culture (*N*)	
Aerobic/Anaerobic/Fungi	3/0/0

ADA, adenosine deaminase; *N*, number; SD, standard deviation; Ser‐H, serum‐hemorrhagic.

a There were 10 cases with insufficient data for classification.

**Figure 4:**
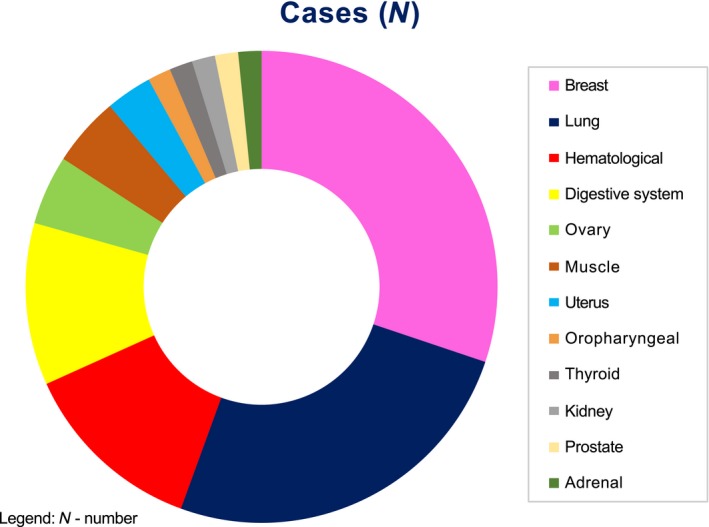
Tumor primary sites in cases with malignant effusion.

In malignant effusions, the most common tumor primary sites were breast and lung, with less representation of the other sites (Fig. 4). Hematological malignancies were represented by seven cases of lymphoma and one case of multiple myeloma. Of the benign effusions, three cases were cardiovascular system diseases, two were tuberculosis, one was cirrhosis, and one was chronic kidney disease.

**Figure 5:**
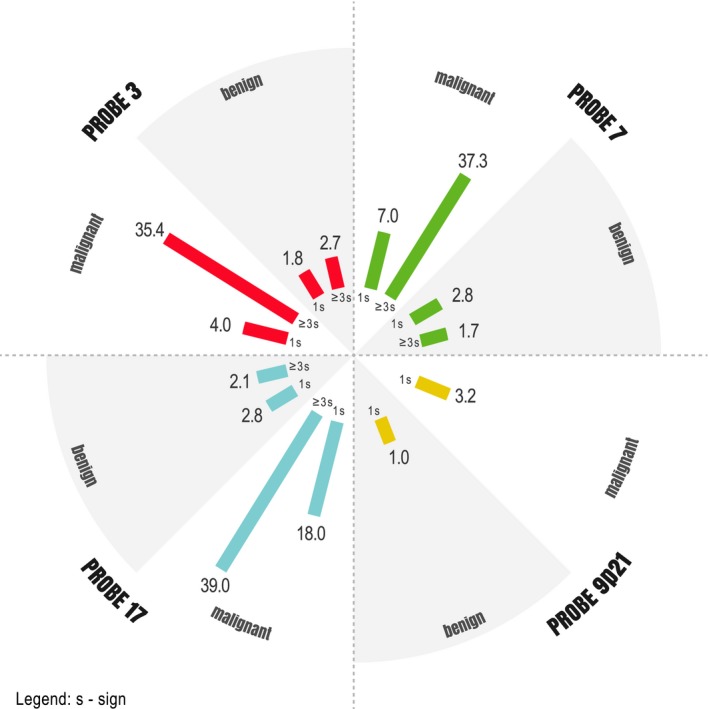
Frequency of cell signals according to probe (median).

In most cases of aneuploidy, a predominance of chromosomal gains was observed. In all cases, it was possible to count 200 cells/case. The signal frequencies for each probe in the *P‐FISH* analysis are shown in Figure 5.

The performance of cytology—considered the gold standard for etiological diagnosis—and FISH (with proposed and manufacturer cutoffs) is shown in Tables 2, 3, 4.

**Table 2:** Diagnostic performance of cytology and *UroVysion* ^*®*^ FISH

	TP (*N*)	TN (*N*)	FP (*N*)	FN (*N*)
Cytology	54	6	1	9
*M‐FISH*	36	6	1	27
*P‐FISH*	55	5	2	8

FN, false negative; FP, false positive; *N*, number; TN, true negative; TP, true positive.

**Table 3:** Sensitivity, specificity, PPV, NVP, and accuracy of cytology and *UroVysion* ^*®*^ FISH

	S (%)	E (%)	PPV (%)	NPV (%)	ACU (%)
Cytology	85.7	85.7	98.1	40.0	85.7
*M‐FISH*	57.1	85.7	97.2	18.1	60.0
*P‐FISH*	87.3	71.4	96.4	38.4	85.7
Cytology and *M‐FISH*	81.3	83.3	97.2	38.4	81.6
Cytology and *P‐FISH*	88.0	83.3	98.1	41.0	87.8

ACU, accuracy; E, specificity; NPV, negative predictive value; PPV, positive predictive value; S, sensitivity.

**Table 4:** Comparative analysis between cytology and *UroVysion* ^*®*^ FISH

Test	*P* a
Cytology vs. *M‐FISH*	<0.0006
Cytology vs. *P‐FISH*	NS
*M‐FISH* vs. *P‐FISH*	<0.0006

NS, not significant.

a *P *<* *0.05 significant.

## Discussion

In the present study, *UroVysion* ^*®*^ FISH using the manufacturer's *cutoff* performed worse than with the proposed *cutoff* for the identification of malignant effusions. Using the proposed *cutoff*, the diagnostic sensitivity was 87.3%, with an accuracy of 85.7%, a PPV of 96.4%, and a specificity of 71.4%. When combined with cytology, the sensitivity was 88.0%, with an accuracy of 87.8%, and an improved specificity of 83.3%. *P‐FISH* was the only examination that identified two cases of malignant pleural effusion secondary to ovarian and lung cancer. However, it failed to identify a case of malignant pleural effusion due to myelomatous infiltration.

The clinical presumption of malignant effusion in patients with or without prior history of cancer poses a challenge to the cytologist, especially in cases where cytology, although atypical, is inconclusive. Thus, complementary examinations of the pleural or peritoneal fluid should be considered potential tools for diagnosis without adding further risk to the patient. In this context, new methodologies such as proteomic assays have shown promising results (for example, *CARD9—isoform 1 of caspase recruitment domain member 9*), but have limitations and are not widely used 19. In the same way, the detection of circulating tumor cells (CTCs) in liquid biopsies has emerged as a tool with great diagnostic and prognostic potential, spawning new clinical trials using blood, urine, saliva, feces, sputum, cavitary, and cerebrospinal fluid for the diagnosis and monitoring of patients with cancer, mainly by detecting copy number variation of genes by next‐generation sequencing (NGS) or FISH assay 20.

The FISH assay is widely used in the clinical laboratory and can be applied in the evaluation of chromosomal abnormalities in nondividing cells, with results available within 24 h 21. In the present study, this cytogenetic technique was used to recognize numerical DNA alterations in genomic regions of interest for oncology without significantly altering the turnaround time (TAT) to diagnosis.

**Figure 6:**
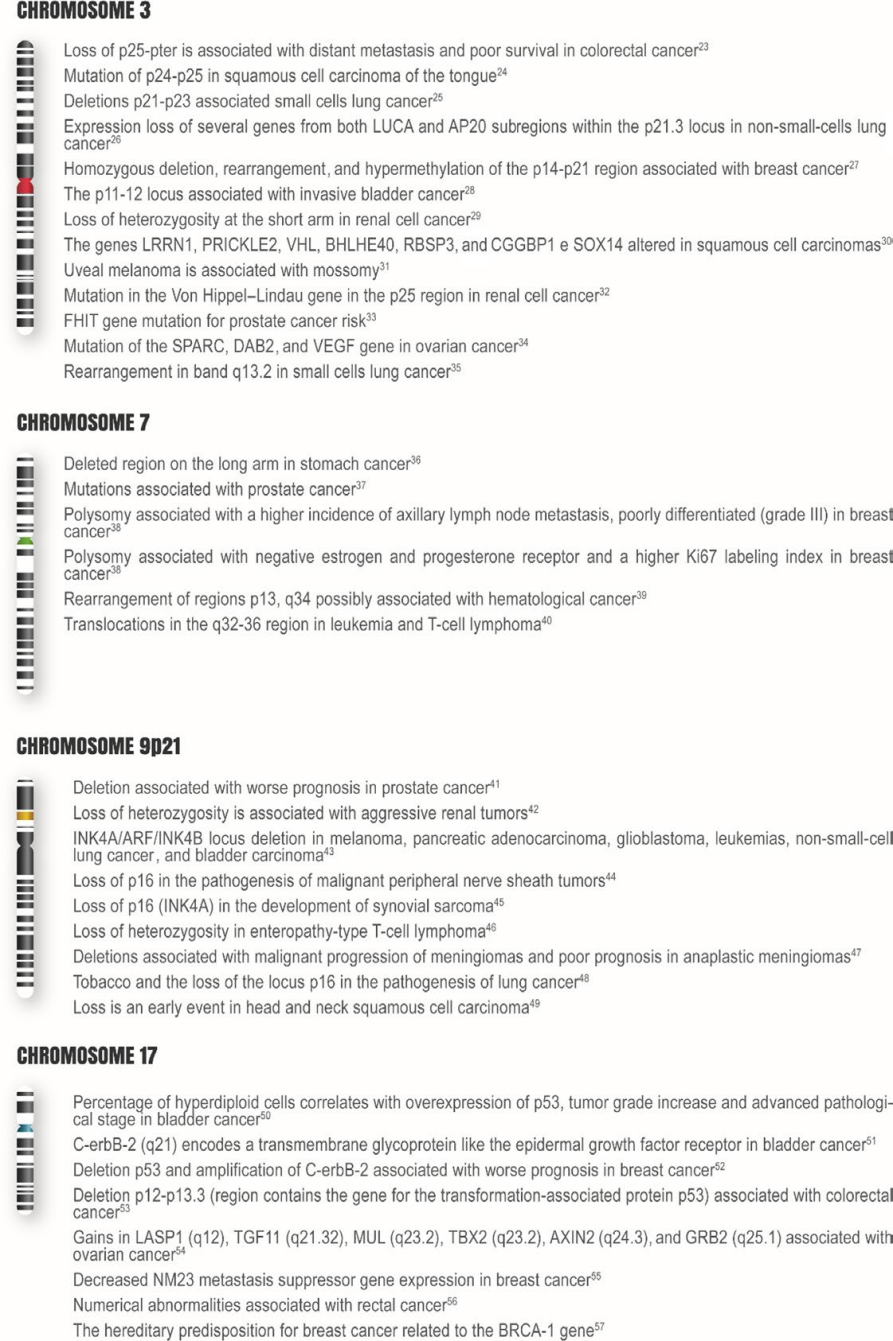
Chromosomal abnormalities observed on chromosomes 3, 7, 17, and 9p21.

Aneuploidy is triggered by a high rate of single chromosomal missegregation, as seen in the chromosomal instability and inactivation of the p53 pathway 11. In cavity fluid, abnormal cellular DNA content may be an important indicator of malignancy, especially in cases in which cytology does not allow for definitive diagnosis 22. In our study, we evaluated the detection of aneuploid cells in effusions with the *UroVysion* ^*®*^ FISH test, which was originally developed for the urine diagnosis and follow‐up of patients with bladder cancer. The *UroVysion* ^*®*^ FISH test consists of four labeled probes that hybridize to the centromeric regions of chromosomes 3, 7, and 17, as well as to the 9p21 locus, which are considered potential targets for carcinogenesis and the development of metastasis 23, 24, 25, 26, 27, 28, 29, 30, 31, 32, 33, 34, 35, 36, 37, 38, 39, 40, 41, 42, 43, 44, 45, 46, 47, 48, 49, 50, 51, 52, 53, 54, 55, 56, 57. Figure 6 details the major chromosomal abnormalities present in these target regions.

Although we obtained satisfactory results with the *UroVysion* ^*®*^ test in the diagnosis of malignant effusions, data in the literature show varying sensitivity, specificity, PPV, and NPV values in the diagnosis and follow‐up of patients with urinary tract tumors (mainly bladder cancer) and of patients with tumors in the bile ducts and pancreas, as can be observed in Table 5. Flores‐Staino et al. 17. reported chromosomal aberrations in 29 samples of pleural fluid from patients with metastatic carcinoma using the *UroVysion* ^*®*^ test. Cora et al. 22, Ioakim‐Liossi et al. 58, Roka et al. 59, and Fiegl et al. 60 also used the FISH assay to investigate chromosomal aberrations in cavitary fluids using other protocols and probes for different chromosomes.

**Table 5:** Literature data describing the use of *UroVysion* ^*®*^ FISH in several cancer types

	Tumor	*N*	S (%)	E (%)	PPV (%)	NPV (%)
Liew et al. 61	Cholangiocarcinoma	30	84.2	100	100	65.4
Virk et al. 62	Bladder	377	44.6	81.8	47.1	80.2
Lavery et al. 63	Bladder	129	67	76	—	—
Gomella et al. 64	Upper Urinary Tract/Bladder	415	50/51.9	69.9/89.3	40.3/90	77.4/50
Gopalakrishna et al. 65	Bladder	2040	37	84	—	—
Mischinger et al. 66	Bladder	1048	71.9	69.3	39.4	89.9
Miki et al. 67	Bladder	91	62.5	100	100	85.7
Zhou et al. 68	Bladder	1532	78.9/65.9^a^	59.2/78.9^a^	77/84.4^a^	61.8/57.1^a^
Dudley et al. 69	Periampullary	72	55	94	—	—
Glass et al. 70	Urothelial	942	55.1	78.7	—	—
Fritcher et al. 71	Cholangiocarcinoma/Pancreas	272	46	91	—	—
Fritsche et al. 72	Urothelial high‐grade	210	95	93	76	99
Breen et al. 73	Urothelial	939	47.7	87.7	—	—
Todenhöfer et al. 74	Bladder	483	74.3	69.6	46.8	88.2
Vlajnic et al. 75	Pancreas and Biliary Pathways	90	26.7	100	100	63.3
Ho et al. 76	Urothelial	627	89.2	83.4	47.1	97.9
Dimashkieh et al. 77	Bladder	1835	61.9	89.7	53.9	87.5
Todenhöfer et al. 77	Upper Urinary Tract/Bladder	2365	70.8/61.5	80.1/80.1	—	—
Youssef et al. 78	Urothelial	123	23.5	94.3	40	88.5
Caraway et al. 79	Urothelial	1006	61	58	42	79
Mian et al. 80	Upper Urinary Tract	55	100	89.5	84.6	100
Kehinde et al. 81	Bladder	178	80	48	61	71.2

E, specificity; *N*, number; NPV, negative predictive value; PPV, positive predictive value; S, sensitivity.

a Criteria considered with tetrasomy/without tetrasomy.

Considering that the majority of patients with malignant effusions present advanced disease with significant systemic impairment 4, the possibility of establishing a cancer diagnosis using aspirated fluid samples is of great value in clinical practice, as it avoids submitting patients to invasive diagnostic procedures. In this study, the increase in diagnostic sensitivity with *P‐FISH* was not significant when compared to cytology. However, the assay was effective in demonstrating aneuploidy and, therefore, in confirming malignancy in all cases of suspicious cytology (14 cases). It is important to emphasize that this study is the first to propose a different *cutoff* value for the diagnosis of malignant pleural or peritoneal effusion with the *UroVysion* ^*®*^ test, including samples from patients with metastases of solid tumors and hematological malignancies.

However, although the results are promising, we must highlight some limitations of the study: (1) the absence of malignant mesothelioma in the study casuistic, as the homozygous deletion of the 9p21 gene is more frequently observed in this type of tumor; (2) the small number of benign effusions, which are important for validation of assay specificity; and (3) the small number of hematological malignancy cases included.

In conclusion, the present study showed that the *UroVysion* ^*®*^ *P‐FISH* was effective in the identification of aneuploid cells in cavity fluids of patients with malignant effusions. *UroVysion* ^*®*^ *P‐FISH* exhibited good sensitivity and accuracy, especially in cases of inconclusive cytology. However, for use in clinical practice, a greater number of effusions should be evaluated, including a wider spectrum of malignancies known to evolve with cavitary effusions.

## Conflict of Interest

The authors declare no conflict of interests.
